# Neurological Complications in Surgical Patients with Left-Sided Infective Endocarditis: Risk Factors, Prognosis, and Surgical Timing

**DOI:** 10.3390/jcdd13010013

**Published:** 2025-12-24

**Authors:** Zining Wu, Jun Zheng, Qi Miao, Shangdong Xu, Guotao Ma, Xingrong Liu, Jianzhou Liu, Sheng Yang, Yanxue Zhao, Xinpei Liu, Chaoji Zhang

**Affiliations:** Department of Cardiac Surgery, Peking Union Medical College Hospital, Chinese Academy of Medical Sciences, Beijing 100730, China; zhengjun@pumch.cn (J.Z.);

**Keywords:** infective endocarditis, neurological complications, valve surgery, prognosis

## Abstract

Background: The aim of this study was to explore the baseline characteristics, risk factors, and prognosis of surgical patients with left-sided valvular infective endocarditis (IE) complicated by preoperative neurological complications, as well as the impact of complication subtypes and surgical timing on outcomes. Methods: A retrospective analysis of 605 consecutive surgical patients with left-sided valvular IE (May 2012–June 2024) was performed. Patients were stratified into neurological complication and non-complication groups, with 1:1 propensity score matching (PSM) balancing baseline confounders. Six neurological complication subtypes were defined; surgical timing was categorized as early (≤7 days for infarction, ≤30 days for hemorrhage) or delayed. Logistic/Cox regression analyzed risk factors and prognosis; subgroup analyses compared modified Rankin Scale (mRS) scores, and Kaplan–Meier curves evaluated long-term survival. Results: Mitral valve involvement, highly mobile vegetations, and longer IE symptom-to-surgery time were risk factors for neurological complications. After PSM balancing, the neurological complications group had similar in-hospital, long-term mortality to the control group, but a significantly higher new-onset cerebral complication rate. In total, 81.5% of complication patients achieving mRS ≤ 2 (good functional status) with infarction showed improved postoperative mRS scores. Cerebral hemorrhage was an independent predictor of in-hospital mortality, while cerebral hemorrhage and regional infarction were independent predictors of new-onset cerebral complication. Early surgery in infarction patients increased the neurological complication rate. Conclusion: Neurological complication incidence was 27.8%. Mitral valve involvement, high vegetation mobility, and preoperative emboli were risk factors. Except for preoperative cerebral hemorrhage and regional infarction, which increase the risk of in-hospital mortality, neurological complications overall do not affect short-term and long-term mortality rates, but increase the risk of postoperative neurological deterioration. Individualized surgical timing is recommended.

## 1. Introduction

Infective endocarditis (IE) remains a life-threatening condition with substantial morbidity and mortality, largely attributed to extracardiac complications [1,2]. Neurological complications are the most common extracardiac manifestations of IE, occurring in 20–40% of cases, and significantly impact clinical decision-making and long-term outcomes [2,3]. These complications are predominantly ischemic, with silent cerebral embolism detected in 60–80% of asymptomatic patients via systematic neuroimaging [2,4]. Left-sided valvular IE is particularly prone to neurological complications due to the direct anatomical pathway of cardiac vegetations to the cerebral circulation [5].

Previous studies have identified vegetation characteristics (size, mobility) and valve involvement as potential risk factors for neurological complications [6,7]. However, the prognostic heterogeneity among different neurological subtypes remains unclear, and direct comparisons between patients with and without neurological complications are often confounded by baseline differences [8].

Surgical timing for IE patients with neurological complications is a core clinical dilemma. The 2023 ESC Guidelines for the Management of Endocarditis provide specific recommendations: urgent surgery (3–5 days) is indicated after transient ischemic attack (TIA) or ischemic stroke in the presence of heart failure, uncontrolled infection, abscess, or persistent embolic risk (Class I, Level B), while a delay beyond 1 month is recommended for intracranial hemorrhage (Class IIa, Level C) [2]. However, these guidelines lack detailed stratification by complication severity, and real-world data on individualized timing are limited [9,10]. Recent studies, such as Lee et al. [9] and Scheggi et al. [10], have explored surgical outcomes in IE patients with stroke or septic cerebral embolism, but the optimal timing for different neurological subtypes remains controversial.

Against this background, this study aimed to: (1) investigate the incidence and baseline characteristics of preoperative neurological complications in left-sided valvular IE surgical patients; (2) compare outcomes of different neurological complications using propensity score matching (PSM) to address baseline confounding; (3) explore the prognostic impacts of neurological complication subtypes; and (4) propose individualized surgical timing strategies aligned with current guidelines. The findings are expected to supplement existing evidence and provide actionable guidance for clinical practice.

## 2. Materials and Methods

### 2.1. Study Subjects and Inclusion/Exclusion Criteria

Inclusion Criteria: Met modified Duke criteria for definite IE [11]; Preoperative cardiac ultrasound confirmed left-sided valve involvement; Underwent valve surgery for indications (uncontrolled infection/heart failure, embolism prevention); Complete data (baseline, neuroimaging, surgery, follow-up).

Exclusion Criteria: Isolated right-sided valvular IE; Comorbid central nervous system diseases (e.g., brain tumors, chronic stroke sequelae).

### 2.2. Data Collection and Definitions

Baseline Data: Demographic information (age, gender), medical history (hypertension, diabetes, structural heart disease), microbiological results, echocardiographic parameters (valve involvement, vegetation size/mobility, periannular lesions), and laboratory indices were retrieved from electronic medical records. Structural heart disease was defined as pre-existing valvular lesions documented before IE onset, excluding valve dysfunction secondary to IE [12]. Preoperative unstable vital signs were defined as the need for non-invasive ventilator support/tracheal intubation with mechanical ventilation, intravenous vasoactive agent infusion/intensive diuretic therapy, or admission to the intensive care unit (ICU) before surgery. Preoperative embolic events were defined as infective endocarditis (IE)-related embolic events excluding cerebral embolism, including renal, splenic, gastrointestinal, limb arterial and cutaneous embolism identified via symptoms and examinations. Highly mobile vegetations were defined as a semi-quantitative mobility score ≥ 3 (1 = fixed to valve, 2 = mild mobility within valve plane, 3 = moderate mobility beyond valve plane, 4 = severe mobility with free movement in cardiac chamber) based on TOE assessments [2,13]. Neurological Complications: Defined based on preoperative clinical symptoms (limb weakness, disturbance of consciousness) and neuroimaging (brain CT/MRI, CTA/MRA). Six subtypes were classified [6,14]: (1) Small ischemic lesions: <15 mm on MRI-DWI, National Institutes of Health Stroke Scale (NIHSS) = 0; (2) Regional cerebral infarction: ≥30% of a lobe/multiple lobes involved or NIHSS ≥ 1; (3) Cerebral hemorrhage: >10 mm intracranial bleeding (CT/MRI); (4) Cerebral microbleeds: 2–10 mm low-signal on MRI-SWI/T2, asymptomatic; (5) Infectious aneurysms: ≥3 mm intracranial artery dilatation (CTA/MRA/DSA) with IE infection evidence; (6) Brain abscess: Imaging and deficit-confirmed intracranial abscess.

Surgical Timing: Defined as the interval from neurological symptom onset/imaging confirmation to surgery: early surgery = ≤7 days for infarction, ≤30 days for hemorrhage; delayed surgery = >7 days for infarction, >30 days for hemorrhage [2,9]. Outcome Indicators: In-hospital mortality, new postoperative cerebral complications (imaging-confirmed ischemic/hemorrhagic lesions), discharge mRS (0 = no symptoms to 6 = death; ≤2 = good functional status), ICU stay duration, renal failure (need for hemodialysis), low cardiac output, multiple organ failure, and long-term survival (up to July 2025).

### 2.3. Statistical Methods

All analyses were conducted using R 4.3.0. (R Foundation for Statistical Computing, Vienna, Austria) Continuous variables were expressed as mean ± SD (*t*-test) or median (IQR) (Mann–Whitney U test); categorical variables as n (%) (chi-square/Fisher’s exact test). Propensity Score Matching (PSM): 1:1 nearest-neighbor matching was performed to balance 6 baseline confounders with *p* < 0.05 difference between groups. Standardized mean differences (SMD < 0.25) indicated successful matching. Univariate and Multivariate Analyses: Variables with *p* < 0.05 in univariate analysis were included in multivariate Logistic regression to identify risk factors for neurological complications and in-hospital mortality. Subgroup Analyses: Comparisons of outcomes among different surgery timings among neurological complications groups were performed. Wilcoxon signed-rank test compared pre-/postoperative mRS in the complication group. Kaplan–Meier curves (Log-rank test) evaluated long-term survival. *p* < 0.05 was statistically significant.

## 3. Results

### 3.1. Characteristics of Patients

Among the 605 surgical patients with left-sided IE, 168 cases (27.8%) were complicated with neurological complications (complication group), and 437 cases (72.2%) had no neurological complications (non-complication group). A total of 168 cases of cerebral complications were identified, including 74 cases of small ischemic lesions, 77 cases of regional cerebral infarction, 57 cases of cerebral hemorrhage, 21 cases of cerebral microbleeds, 12 cases of cerebral aneurysms, and 21 cases of cerebral abscesses. Some patients had multiple concurrent complications, so the sum of individual subtypes exceeds the total number of cases. A comparison of baseline characteristics between the two groups before and after PSM is presented in Table 1. The complication group exhibited a higher proportion of mitral valve involvement, a lower proportion of aortic valve involvement, a higher prevalence of Structural heart disease (valvular heart disease or congenital heart disease), a greater proportion of vegetations with high mobility, a higher incidence of preoperative embolic events, and higher Carlson scores (all *p* < 0.05). A total of 71 patients (11.7%) presented with preoperative unstable vital signs, among whom 25 required non-invasive ventilator support or tracheal intubation with mechanical ventilation, 31 received intravenous vasoactive agent infusion, and 40 were admitted to the ICU. Propensity score matching yielded a matched cohort of 192 patients, with balanced baseline characteristics between the two groups in this cohort (Table 1).

### 3.2. Risk Factors for Preoperative Neurological Complications

Univariate analysis revealed that mitral valve involvement, high mobility of vegetations, a history of embolic events, and the time interval from the onset of IE symptoms to surgery were significantly associated with the development of neurological complications (*p* < 0.05) (Table 2). Variables that were statistically significant in univariate analysis failed to be incorporated into a valid multivariate analysis model.

### 3.3. Comparison of Short-Term and Long-Term Prognosis

The median follow-up duration for the 605 patients was 52 months. In-hospital mortality occurred in 26 patients (4.3%), including 15 cases (3.4%) in the no cerebral complications group, 11 cases (6.6%) in the cerebral complication group, 6 cases (10.5%) in the cerebral hemorrhage group, 7 cases (9.1%) in the regional cerebral infarction group, 1 case (4.8%) in the cerebral microbleeds group, 0 cases (0%) in the infectious aneurysms group, 3 cases (4.1%) in the small ischemic lesions group, and 1 case (4.8%) in the brain abscess group. A total of 72 patients (12.1%) died by the follow-up endpoint, among whom 51 cases (11.7%) were in the no cerebral complications group, 22 cases (13.1%) in the cerebral complication group, 11 cases (19.3%) in the cerebral hemorrhage group, 13 cases (16.9%) in the regional cerebral infarction group, 1 case (4.8%) in the cerebral microbleeds group, 0 cases (0%) in the infectious aneurysms group, 8 cases (10.8%) in the small ischemic lesions group, and 5 cases (23.8%) in the brain abscess group. In unmatched cohort, the in-hospital mortality was higher in the complication group than in the non-complication group, the difference did not reach statistical significance (6.6% vs. 3.4%, *p* = 0.09). In matched cohort, there was no significant difference in in-hospital mortality and long-term mortality between the two groups. Notably, the incidence of new-onset cerebral hemorrhage and new-onset cerebral infarction is higher in the complication group after surgery (both *p* < 0.05). The results of the matched cohort maintained this difference. There was no significant difference in the incidence of adverse events (such as postoperative implantation of intra-aortic balloon pump, extracorporeal membrane oxygenation, continuous renal replacement therapy, permanent pacemaker implantation, low cardiac output syndrome, and multiple organ failure) or in the length of ICU stay between the two groups, whether in the unmatched or matched cohorts (Table 3).

In the matched cohort, there was no significant difference in in-hospital and long-term mortality rates between the cerebral hemorrhage group and no complication group, but the incidence of new cerebral complications after surgery was significantly increased. There was no significant difference in in-hospital and long-term mortality rates between the cerebral infarction group and no complication group, but the incidence of new cerebral complications after surgery was significantly increased. (Supplementary Table S1).

**Figure 1:**
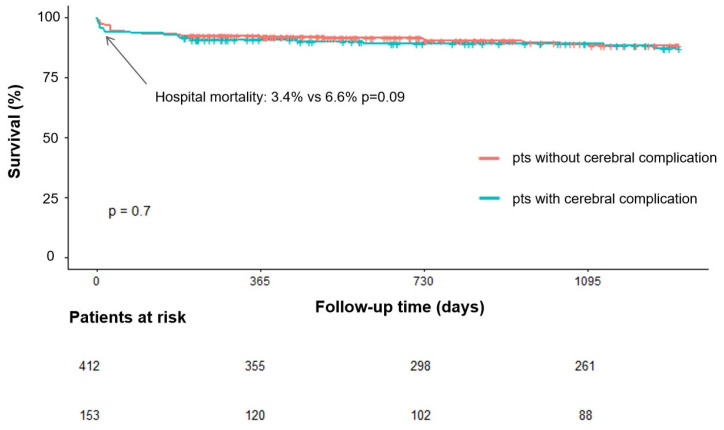
Long-term survival in patients with cerebral complication compared with patients without cerebral complication.

Kaplan–Meier analysis indicated no significant difference in survival curves between the complication group and the non-complication group (Log-rank *p* = 0.7) (Figure 1). Comparing the survival curves of cerebral infarction (Log-rank *p* = 0.06)/cerebral hemorrhage (Log-rank *p* = 0.183) and no cerebral complication groups separately, it was found that there was no statistical difference between the groups.

### 3.4. Association of Neurological Complications with Adverse Prognosis

Univariate analysis found that cerebral hemorrhage, regional cerebral infarction, gender, staphylococcus aureus infection, prosthetic valve endocarditis (PVE), exogenous implants, pancytopenia, NYHA class III–IV, aortic absence, LVEF, and cardiopulmonary bypass time are associated with in-hospital mortality (Supplementary Table S2). Cerebral hemorrhage, pancytopenia, heart failure, aortic annular abscess, NIHSS score, and mRS score were found to be associated with postoperative new brain complications in univariate analysis (Supplementary Table S3). After adjusting for these confounding factors, respectively, both cerebral hemorrhage (OR = 4.65) and regional cerebral infarction (OR = 3.94) were confirmed as independent risk factors for in-hospital mortality. Cerebral hemorrhage (OR = 6.87) was an independent risk factor for new-onset cerebral complications after surgery (Table 4). Cox regression analysis for long-term mortality demonstrated that none of the various neurological complications exhibited a significant risk effect in the univariate analysis.

### 3.5. Changes in Preoperative and Postoperative mRS Scores in the Complication Group

**Figure 2:**
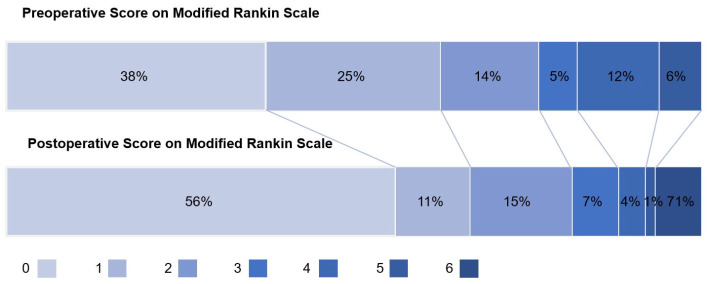
Distribution of modified Rankin scale scores for cerebral complication group’s before surgery and at discharge; Scores range as follows: 0 points (no symptoms), 1 point (mild symptoms without impairment of daily activities), 2 points (mild disability with the ability to walk independently), 3 points (moderate disability requiring assistance for ambulation), 4 points (severe disability with inability to perform self-care), 5 points (severe disability necessitating long-term care), and 6 points (death).

All patients in the complication group completed preoperative and discharge mRS assessments (Figure 2). The median postoperative mRS score was significantly lower than the preoperative score (0 vs. 1, t = 2.90, *p* = 0.004), and 81.5% of patients achieved a postoperative mRS score of ≤2. Among the different types of complications, patients with small ischemic lesions and cerebral microbleeds demonstrated the most favorable recovery (median postoperative mRS score: 0), while patients with cerebral hemorrhage, regional cerebral infarction, and brain abscess exhibited relatively slower recovery (median postoperative mRS score: 1) (Table 5).

### 3.6. Impact of Surgical Timing on Prognosis

**Figure 3:**
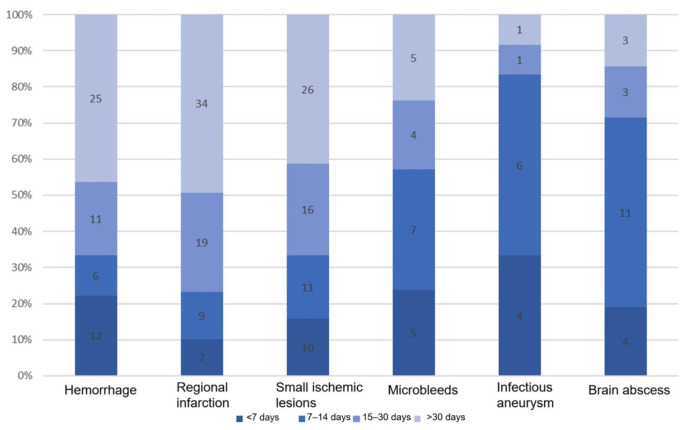
Distribution of surgical timing for different types of cerebral complications.

The time interval from the onset of neurological symptoms or imaging confirmation of lesions to surgery for different types of cerebral complications is presented in Figure 3. The median time interval was 26 days for intracerebral hemorrhage, 29 days for regional cerebral infarction, 23 days for small ischemic lesions, 13 days for microbleeds, 10 days for infectious aneurysms, and 11 days for cerebral abscesses. As illustrated, patients with cerebral hemorrhage or cerebral infarction exhibited a significantly longer surgical delay compared to those with other complication types. Subgroup analysis indicated that patients with cerebral hemorrhage or cerebral infarction who underwent early surgery had higher in-hospital mortality rates, although the differences between the groups were not statistically significant. Notably, patients with cerebral infarction who underwent early surgery had a significantly increased risk of new-onset cerebral hemorrhage (40% vs. 10.4%) and new-onset cerebral infarction (20% vs. 3%). In the regional cerebral infarction group, 40% of patients received early surgery had unstable vital signs, whereas this proportion was 10.2% among those who received delayed surgery. No significant differences in short-term prognosis were observed between patients with small ischemic lesions, microbleeds, aneurysms, or brain abscesses who underwent early surgery and those who did not (Table 6).

## 4. Discussion

### 4.1. Incidence and Risk Factors

The incidence of preoperative neurological complications was 27.8%, consistent with Arregle et al. [6] (26.8%) and within the 20–40% range specified in the 2023 ESC Guidelines [2]. This consistency confirms that neurological complications are a common comorbidity in left-sided valvular IE surgical patients. Mitral valve involvement, highly mobile vegetations, and longer symptom-to-surgery time were identified as independent risk factors. These findings align with the 2023 ESC Guidelines, which emphasize vegetation size and mobility as key predictors of embolic risk [2]. The association between mitral valve involvement and neurological complications, despite the higher hemodynamic velocity of aortic vegetations, is explained by the anatomical location of mitral vegetations, which are more prone to fragmentation and embolization to the cerebral circulation [15].

### 4.2. Prognostic Heterogeneity of Neurological Subtypes

Contrary to prior studies [9,10,16], our surgical cohort’s PSM-matched analysis showed no significant in-hospital/long-term mortality differences between neurological complication (overall, cerebral hemorrhage, infarction) and non-complication groups. This likely reflects the relative safety of timely surgery for IE with neurological complications, avoiding mortality risks from delayed intervention (e.g., recurrent embolism, uncontrolled infection) [2,6]. Notably, cerebral hemorrhage remained an independent in-hospital mortality predictor in multivariable regression (aOR = 4.65, 95% CI: 2.13–10.17, *p* < 0.001)—aligning with its inherent prognostic risk [16]. In fact, if all infective endocarditis (IE) patients undergo MRI, up to 70% will have neurological abnormalities [17], most of which are asymptomatic silent cerebral complications. This high prevalence means neurological complications as a whole cannot be regarded as a high-risk group; thus, neurological abnormalities must be subdivided into distinct subgroups, and their prognostic impacts should be evaluated within each subgroup.

Neurological subgroups had significantly higher postoperative new cerebral complications in the matched cohort. While timely surgery mitigates mortality, cerebral hemorrhage’s prognostic role and elevated neurodeterioration risk warrant targeted perioperative monitoring [2,18,19]—consistent with 2023 ESC Guidelines [2] and complementing Sara et al.’s [16] 2024 data.

### 4.3. Surgical Timing Optimization

The 2023 ESC Guidelines recommend urgent surgery for ischemic stroke with heart failure/uncontrolled infection/abscess/high embolic risk (Class I, Level B) and delayed surgery (>1 month) for intracranial hemorrhage (Class IIa, Level C) [2]. Our findings support these recommendations and provide additional granularity. Early surgery showed a trend toward increased postoperative neurological deterioration, but the difference was not statistically significant. This aligns with the guideline’s emphasis on individualized decision-making, balancing the risk of delayed surgery (embolism, cardiac deterioration) and early surgery (neurological deterioration) [2,6]. The finding that delayed surgery for cerebral hemorrhage was associated with lower mortality is consistent with Lee et al. [9], who reported improved outcomes with delayed surgery for preoperative intracranial hemorrhage. Of note, patients undergoing early surgery tend to have hemodynamic instability, and other urgent conditions, early surgery helps eliminate valvular regurgitation and prevent circulatory collapse. Therefore, for IE patients meeting emergency surgical indications, optimal surgical timing should not be missed due to fear of postoperative neurological complication risks.

### 4.4. Neurological Functional Prognosis

A notable finding of this study is the favorable neurological functional outcome in patients with preoperative neurological complications: the median postoperative modified Rankin Scale (mRS) score improved from preoperative levels (median: 2) to 1, with 81.5% of patients achieving mRS ≤ 2 (good functional status). The prior literature predominantly focused on mortality and rarely reported detailed postoperative neurological functional recovery—creating a knowledge gap in assessing surgical safety for IE patients with neurological complications [6,9,10,16]. This favorable functional outcome confirms that timely cardiac surgery for left-sided valvular IE with neurological complications is neurologically safe, beyond mitigating mortality risks from delayed intervention. Several mechanisms may explain this result: first, surgical removal of highly mobile vegetations eliminates the source of recurrent embolism, preventing further neurological damage [2,6]; second, standardized perioperative neuroprotective management (e.g., strict blood pressure control, antiplatelet therapy when indicated) minimizes secondary brain injury [14]; third, the younger cohort has better neurological plasticity, facilitating recovery from preoperative lesions [20]. Notably, even patients with regional infarction showed meaningful functional improvement, which has rarely been reported in previous surgical cohorts [9,16].

### 4.5. Limitations

Retrospective design with inherent selection bias; MRI was not systematically used in the early study period, potentially underestimating small ischemic lesions; the young cohort may limit generalizability to older populations; lack of data on long-term cognitive outcomes, requiring confirmation in larger prospective studies.

## 5. Conclusions

The incidence of preoperative neurological complications in left-sided valvular infective endocarditis (IE) surgical patients was 27.8%. Mitral valve involvement, highly mobile vegetations, delayed surgery and preoperative emboli were confirmed as risk factors. Notably, while cerebral hemorrhage and regional cerebral infarction remained independent predictors of in-hospital mortality, propensity score-matched analysis showed no significant differences in short-term or long-term survival between patients with neurological complications and those without—supporting the safety of timely surgery in this cohort by avoiding delayed intervention-related risks. However, the complication group had a significantly higher rate of new postoperative cerebral complications. A favorable novel finding was the improved neurological functional outcome even for severe subtypes. Individualized surgical timing is recommended aligned with 2023 ESC Guidelines. As a whole, neurological complications do not affect survival rates in IE surgical patients, but intracerebral hemorrhage and regional cerebral infarction can be identified as high-risk subgroups. This study provides novel evidence for the neurological safety of surgery, reinforcing the rationale for timely intervention in eligible patients.

## Figures and Tables

**Table 1:** Comparison of baseline characteristics of patients.

		Before PSM			After PSM		
	Total Population (*n* = 605)	Non-Complication Group (*n* = 437)	Complication Group (*n* = 168)	*p* Value	Non-Complication Group (*n* = 96)	Complication Group (*n* = 96)	*p* Value
Age	49.00 (36.00–59.00)	51.00 (37.00–59.00)	46.50 (35.00–57.25)	0.182	47.00 (33.00, 57.25)	44.00 (33.75, 56.00)	0.586
Male	427 (70.58%)	312 (71.40%)	115 (68.45%)	0.477	62 (64.58%)	61 (63.54%)	0.880
Underlying diseases							
Hypertension	161 (26.61%)	124 (28.38%)	37 (22.02%)	0.113	25 (26.04)	20 (20.83)	0.394
Diabetes mellitus	56 (9.26%)	43 (9.84%)	13 (7.74%)	0.424	7 (7.29)	8 (8.33)	0.788
Coronary heart disease	72 (11.90%)	53 (12.13%)	19 (11.31%)	0.781	10 (10.42)	10 (10.42)	1.000
Atrial fibrillation	35 (5.79%)	27 (6.18%)	8 (4.76%)	0.504	4 (4.17)	7 (7.29)	0.352
Structural heart disease	394 (65.12%)	295 (67.51%)	99 (58.93%)	0.047	63 (65.62)	59 (61.46)	0.549
Exogenous implants	56 (9.26%)	42 (9.61%)	14 (8.33%)	0.627	8 (8.33)	9 (9.38)	0.799
Carlson comorbidity score	1(0–2)	1 (0–2)	1 (1–2)	0.003	1.00 (0.00, 2.00)	1.00 (1.00, 2.00)	0.121
Euroscore II (%)	2.40 (1.38, 5.63)	2.40 (1.38, 5.59)	2.42 (1.27, 5.92)	0.931	2.13 (1.32, 5.54)	2.75 (1.62, 5.87)	0.131
Type of IE							
Prosthetic valve IE	49 (8.10%)	36 (8.24%)	13 (7.74%)	0.84	90 (93.75)	87 (90.62)	0.420
Aortic valve	337 (55.70%)	256 (58.58%)	81 (48.21%)	0.022	47 (48.96)	46 (47.92)	0.885
Mitral valve	391 (64.63%)	272 (62.24%)	119 (70.83%)	0.048	66 (68.75)	66 (68.75)	1.000
Bivalvular (aortic + mitral)	167 (27.60%)	127 (29.06%)	40 (23.81%)	0.196	24 (25.00)	23 (23.96)	0.867
Cardiac ultrasound indicators							
LVEF	66 (60–70)	66 (61–70)	66 (60–71)	0.763	67 (61–71)	67 (62–71)	0.769
Vegetation diameter ≥ 10 mm	274 (45.29%)	203 (46.45%)	71 (42.26%)	0.354	48 (50.00)	44 (45.83)	0.563
High mobility of vegetations	198 (32.73%)	131 (29.98%)	67 (39.88%)	0.020	42 (43.75)	41 (42.71)	0.884
Aortic annular abscess	73 (12.07%)	52 (11.90%)	21 (12.50%)	0.839	10 (10.42)	12 (12.50)	0.650
Mitral annular abscess	33 (5.45%)	23 (5.26%)	10 (5.95%)	0.738	4 (4.17)	7 (7.29)	0.352
Etiological results							
Staphylococcus aureus	48 (7.93%)	31 (7.09%)	17 (10.12%)	0.218	9 (9.38)	10 (10.42)	0.809
Streptococcus	279 (46.12%)	204 (46.68%)	75 (44.64%)	0.652	42 (43.75)	35 (36.46)	0.303
Other Gram-positive bacteria	55 (9.09%)	40 (9.15%)	15 (8.93%)	0.931	4 (4.17)	11 (11.46)	0.060
Gram-negative bacteria	38 (6.28%)	26 (5.95%)	12 (7.14%)	0.588	3 (3.12)	6 (6.25)	0.495
Fungi	14 (2.31%)	9 (2.06%)	5 (2.98%)	0.502	3 (3.12)	4 (4.17)	1.000
IE characteristics							
Embolic events	216 (35.70%)	80 (18.31%)	136 (80.95%)	<0.001	65 (67.71)	65 (67.71)	1.000
Refractory fever	165 (27.27%)	121 (27.69%)	44 (26.19%)	0.711	25 (26.04)	25 (26.04)	1.000
NYHA class Ⅲ-Ⅳ	209 (34.55%)	149 (34.10%)	60 (35.71%)	0.708	30 (31.25)	35 (36.46)	0.446
Unstable vital signs	71 (11.74%)	50 (11.44%)	21 (12.50%)	0.717	11 (11.46)	13 (13.54)	0.061
Antibiotic treatment ≥ 7 days	395 (65.29%)	281 (64.30%)	114 (67.86%)	0.411	69 (71.88)	75 (78.12)	0.317

LVEF: Left ventricular ejection fraction; NYHA: New York Heart Association Functional Classification.

**Table 2:** Univariate Analysis of Neurological Complications in Surgical Patients with Left-Sided IE.

	OR (95% CI)	*p* Value
Involved valve (mitral valve)	1.47 (1.00, 2.16)	0.0485
High mobility of vegetations	1.55 (1.07, 2.24)	0.0205
Embolic events	18.97 (12.03, 29.89)	<0.0001
Time from IE symptom onset to surgery (months)	1.05 (1.00, 1.09)	0.0422

**Table 3:** Comparison of Prognosis between Patients Complicated with and without Neurological Complications.

	Unmatched Cohort (*n* = 605)			Propensity Matched Cohort (*n* = 192)		
	No Cerebral Complications (*n* = 437)	Cerebral Complications (*n* = 168)	*p* Value	No Cerebral Complications (*n* = 96)	Cerebral Complications (*n* = 96)	*p* Value
In-hospital death	15 (3.43%)	11 (6.55%)	0.091	4 (4.17%)	5 (5.21%)	1.00
Long-term death	51 (11.67%)	22 (13.10%)	0.529	11 (11.46%)	10 (10.42%)	0.837
New-onset cerebral hemorrhage	5 (1.14%)	17 (10.12%)	<0.001	0 (0%)	7 (7.29%)	0.021
New-onset cerebral infarction	3 (0.69%)	8 (4.76%)	0.003	0 (0%)	7 (7.29%)	0.021
IABP	9 (2.06%)	4 (2.38%)	1.00	3 (3.12%)	2 (2.08%)	1.00
ECMO	10 (2.29%)	1 (0.6%)	0.291	4 (4.17%)	1 (1.04%)	0.365
Permanent pacemaker	13 (2.97%)	2 (1.19%)	0.331	3 (3.12%)	2 (2.08%)	1.00
CRRT	49 (11.21%)	17 (10.12%)	0.699	8 (8.33%)	10 (10.42%)	0.62
MOF	16 (3.66%)	4 (2.38%)	0.43	3 (3.12%)	1 (1.04%)	0.613
LCOS	36 (8.24%)	13 (7.74%)	0.84	7 (7.29%)	7 (7.29%)	1.00
ICU stay (days)	2.00 (1.00, 5.00)	2.00 (1.00, 4.75)	0.999	2.00 (1.00, 5.00)	2.00 (1.00, 4.00)	0.810

IABP: Intra-aortic balloon pump; ECMO: Extracorporeal membrane oxygenation; CRRT: continuous renal replacement therapy; MOF: multiple organ failure; LCOS: low cardiac output.

**Table 4:** Association of neurological complications with adverse prognosis.

	OR	95% CI	*p* Value
Cerebral hemorrhage with hospital death			
unadjusted	3.11	1.19–8.08	0.0202
adjusted for age and sex	3.36	1.25–9.05	0.0163
multivariable adjusted *	4.65	1.38–15.68	0.0132
Regional cerebral infarction with hospital death			
unadjusted	2.68	1.09–6.60	0.0322
adjusted for age and sex	2.96	1.18–7.45	0.0208
multivariable adjusted *	3.94	1.20–12.93	0.0237
Cerebral hemorrhage with new-onset cerebral complications			
unadjusted	10.50	4.70–23.45	<0.001
adjusted for age and sex	10.21	4.52–23.07	<0.001
multivariable adjusted **	6.87	2.48–19.06	<0.001

* Adjusted for gender, Staphylococcus aureus infection, prosthetic valve endocarditis (PVE), exogenous implants, pancytopenia, NYHA class Ⅲ-Ⅳ, aortic annular abscess, LVEF, and cardiopulmonary bypass time; ** Adjusted for pancytopenia, heart failure, aortic annular abscess, NIHSS score, and mRS score.

**Table 5:** Comparison of Preoperative and Postoperative mRS Scores in Patients with Different Types of Neurological Complications.

Type of Complication	Case Number (*n*)	Preoperative mRS Score	Postoperative mRS Score	*p* Value
Overall cerebral complications	168	1 (0–2)	0 (0–2)	0.004
Small ischemic lesions	74	0 (0–1)	0 (0–0)	0.158
Cerebral infarction	77	2 (1–4)	1 (0–2)	0.024
Cerebral hemorrhage	57	2 (1–4)	1 (0–3)	0.132
Cerebral microbleeds	21	1 (0–2)	0 (0–1)	0.101
Infectious aneurysm	12	1 (1–4)	0.5 (0–2.5)	0.146
Brain abscess	21	2 (1–3)	1 (0–2)	0.170

**Table 6:** Prognosis in different surgical timing subgroups.

Type of Complication	Surgical Timing		*p* Value
Cerebral hemorrhage			
	Delayed (>30 days)	Early (≤30 days)	
In-hospital death	1 (3.3%)	5 (18.5%)	0.062
New-onset cerebral hemorrhage	5 (16.7%)	8 (29.6%)	0.244
New-onset cerebral infarction	1 (3.3%)	2 (7.4%)	0.492
Regional cerebral infarction			
	Delayed (>7 days)	Early (≤7 days)	
In-hospital death	5 (7.5%)	2 (20.0%)	0.198
New-onset cerebral hemorrhage	7 (10.4%)	4 (40.0%)	0.013
New-onset cerebral infarction	2 (3.0%)	2 (20.0%)	0.024

## Data Availability

The original data of this study include patient-related factors. Due to privacy concerns, the original data of this study will not be made public. If readers need to obtain the original data, please contact the corresponding author.

## Supplementary Materials

The following supporting information can be downloaded at: https://www.mdpi.com/article/10.3390/jcdd13010013/s1, Table S1: Comparison of prognosis between hemorrhage or infarction and no cerebral complications groups in non matched and Unmatched cohort, Table S2: Univariate Analysis of in-hospital mortality, Table S3: Univariate Analysis of Postoperative Cerebral Complications.

## Abbreviations

The following abbreviations are used in this manuscript:

IE	Infective endocarditis
LVEF	Left ventricular ejection fraction
NYHA	New York Heart Association Functional Classification
mRS	modified Rankin Scale scores
