# Supplementary material for: Neurological Complications in Surgical Patients with Left-Sided Infective Endocarditis: Risk Factors, Prognosis, and Surgical Timing
Source: J Cardiovasc Dev Dis. 2025 Dec 24;13(1):13. doi: 10.3390/jcdd13010013 (PMC12841926; doi:10.3390/jcdd13010013)
Supplement: Supplementary file 1 [file jcdd-13-00013-s001.zip › jcdd-3990478-supplementary.pdf]

Supplymentary Table 1. Comparison of prognosis between hemorrhage or infarction and no cerebral complications groups in non matched and Unmatched cohort

	**Unmatched cohort (n = 605)**			**Propensity matched cohort (n = 192)**		
	**No Cerebral Complications (n=437)**	**Regional Cerebral Infarction Group (n=77)**	**P Value**	**No Cerebral Complications (n=96)**	**Regional Cerebral Infarction Group (n=44)**	**P Value**
In-hospital death	15 (3.43%)	7 (9.09%)	0.050	4 (4.17%)	3 (6.82%)	0.802
Long-term death	51 (12.38%)	113 (17.81%)	0.206	11 (11.46%)	5 (11.36%)	0.987
New-onset cerebral hemorrhage	5 (1.14%)	11 (14.29%)	<0.001	0 (0%)	3 (6.82%)	0.030
New-onset cerebral infarction	6 (1.21%)	4 (5.19%)	0.009	0 (0%)	3 (6.82%)	0.030
	**No Cerebral Complications (n=437)**	**Cerebral Hemorrhage Group (n=57)**	**P Value**	**No Cerebral Complications (n=96)**	**Cerebral Hemorrhage Group (n=28)**	**P Value**
In-hospital death	15 (3.43%)	6 (10.53%)	0.032	4 (4.17%)	3 (10.71%)	0.392
Long-term death	51 (12.38%)	11 (21.15%)	0.080	11 (11.46%)	5 (17.86%)	0.57
New-onset cerebral hemorrhage	5 (1.14%)	13 (22.81%)	<0.001	0 (0%)	5 (17.86%)	<0.001
New-onset cerebral infarction	6 (1.21%)	3 (5.26%)	0.023	0 (0%)	2 (7.14%)	0.050

Supplymentary Table 2. Univariate Analysis of in-hospital mortality

**Variables**	**OR**	**(95%CI)**	**P Value**
Female	2.51	(1.14 ~ 5.53)	0.022

Variables	OR	(95%CI)	P Value
Staphylococcus aureus infection	2.97	(1.07 ~ 8.26)	0.037
PVE	2.90	(1.04 ~ 8.05)	0.042
Exogenous implants	3.17	(1.22 ~ 8.27)	0.018
Pancytopenia	5.27	(2.17 ~ 12.80)	<.001
NYHA class Ⅲ-Ⅳ	6.88	(2.72 ~ 17.41)	<.001
Aortic annular abscess	3.51	(1.47 ~ 8.40)	0.005
LVEF, %	0.95	(0.91 ~ 0.99)	0.036
CPB time, minute	1.01	(1.01 ~ 1.01)	<.001
Cerebral hemorrhage	3.11	(1.19 ~ 8.08)	0.020
Regional cerebral infarction	2.68	(1.09 ~ 6.60)	0.032

Supplymentary Table 3. Univariate Analysis of Postoperative Cerebral Complications

Variables	OR	(95%CI)	P Value
Pancytopenia	3.08	(1.19 ~ 7.96)	0.021
NYHA class Ⅲ-Ⅳ	2.28	(1.06 ~ 4.88)	0.034
Aortic annular abscess	2.58	(1.06 ~ 6.30)	0.037
NIHSS score	1.40	(1.21 ~ 1.61)	<.001
Pre-op mRS score	1.44	(1.10 ~ 1.88)	0.007
Cerebral hemorrhage	10.50	(4.70 ~ 23.45)	<.001
